# Analysis of the retraining strategies for multi-label text message classification in call/contact center systems

**DOI:** 10.1038/s41598-024-60697-0

**Published:** 2024-05-02

**Authors:** Katarzyna Poczeta, Mirosław Płaza, Michał Zawadzki, Tomasz Michno, Maria Krechowicz

**Affiliations:** 1 Faculty of Electrical Engineering, Automatics Control and Computer Science, Kielce University of Technology, 25-314 Kielce, Poland; 2 Faculty of Management and Computer Modelling, Kielce University of Technology, 25-314 Kielce, Poland

**Keywords:** Retraining strategy, Text classification, Call/contact center, Multi-label classification, Computer science, Information technology

## Abstract

Today, in many areas of technology, we can come across applications of various artificial intelligence methods. They usually involve models trained on some specific pool of learning data. Sometimes, however, the data analyzed by these solutions can change its nature over time. This usually results in a decrease in classification efficiency. In such a case, the use of techniques to retrain the originally trained reference models should be considered. One of the industries where the nature of data changes quite dynamically over time is the broadly defined call/contact center systems. An example of a module that is often found in this type of system and that, due to frequently changing marketing campaigns, requires the use of learning techniques is the automatic classification of text data. The paper describes the process of retraining the original reference models used in a multi-label text message classification method dedicated directly to call/contact center systems applications. In order to carry out the retraining process, Polish-language data from the actual archives of a large commercial contact center system and English-language data extracted from a publicly available database were used. The study was conducted for models based on artificial neural networks and bidirectional encoder representations from transformer type models. In addition, two different retraining strategies were studied, the results of which were compared with data obtained from the operation of reference models. As a result of the research work, an improvement of up to 5% in classification efficiency, as described by the metric Emotica was obtained, which means that proper integration of the retraining process brings tangible benefits to the solution tested in the article. Thus, it can also benefit the solutions used in business.

## Introduction

Currently, many solutions in the area of popular Call/Contact Center (CC) systems are developing at a very fast pace^1^. This is due, among other things, to the increasing possibilities of implementing artificial intelligence algorithms in business applications. Current trends in the development of CC systems include a directly dedicated method for transcribing voice calls^2^. This method can, for example, be used in implementations of virtual assistants (bots) operating in the audio channels of CC systems. Also, the technologies for building intelligent bots implemented in both audio (voicebots) and text (chatbots) channels are currently developing extremely rapidly^3^. In addition, emotion recognition methods are a popular research trend, whose widespread implementation in CC systems could significantly improve many of the services offered^4,5^. For very many years, an important component of CC systems has also been methods for classifying messages and text documents^6,7^.

All the above-mentioned solutions make very extensive use of elements of artificial intelligence, especially elements of machine learning (ML), Natural Language Processing (NLP) methods, or Big Data analytics methods. However, these solutions are usually based on models learned from data collected at the design stage of a specific subsystem. It should be emphasized that CC's peculiarities include the great thematic diversity of parallel campaigns (in both audio and text channels). For example, we can mention campaigns related to: the sale of various services, debt collection, advertising campaigns, or contacts related to technical defects or contract negotiations, and many, many others. Due to the wide thematic variation in CC, models prepared based on learning data from a certain subject area may have much less effectiveness when applied to campaigns with related or completely different themes. At the same time, it should be noted that, in many cases, the preparation of a universal model would be a very difficult and sometimes even impossible task. Therefore, in the case of CC systems, an important element in maintaining adequate effectiveness of solutions using artificial intelligence algorithms is to complement them with appropriate retraining methods. The task of these methods should be to continuously and dynamically adjust the reference models used in real-time. Such solutions are used in industrial applications with very limited or no use at all. Therefore, the above issues were the main motivation for the authors to undertake the research described in the paper.

The primary objective of this work was to develop a dedicated solution to the CC industry's significant problems related to the need for dynamic retraining of models of the classification methods used. Among the most popular approaches in the problem of retraining artificial intelligence models are two variants. The first consists of direct retraining of the existing network model using only new data. The second, on the other hand, involves retraining the classifier with the full dataset, updated with newly acquired data^8^. The research and analysis presented in the rest of this article are based on the aforementioned two retraining strategies. The issue of multi-label classification of text messages was chosen as the immediate research area, which also continues the authors' earlier research work^9^. It should be noted that the learning processes considered in the paper, after appropriate modifications, can be used in the development of retraining algorithms for other solutions used in CC systems. The contribution to the body of knowledge of this paper is as follows:

- determination of the methodology for carrying out the processes of retraining the reference models used in the method of multi-label classification of text messages dedicated directly to applications in the CC industry;
- a comparative analysis of the results obtained in terms of models based on artificial neural networks (ANN), Bidirectional Encoder Representations from Transformers (BERT) model type, and two text data classification models dedicated directly to the Polish language: HerBERT and PolBERT;
- the proposed approach is the first solution to the problem of implementing a dynamic retraining process for the multi-label method of content classification so that it can be practically applied regardless of the subject matter of CC campaigns.

The paper presents and compares the results obtained for the following types of models: (1) for a classifier based on ANN^9^, (2) for the HerBERT model^10^, (3) for the PolBERT model^11,12^, and for the BERT model^13^. Experimental results showed that the approach proposed in this paper improves the quality of text message classification compared to the solution in which the retraining process was not implemented. The best classification accuracy for Polish-language data was obtained for the HerBERT model, and for English-language data was obtained for the BERT model; nevertheless, the efficiency of the other models is also satisfactory.

The research questions were formulated as follows:

- RQ1: What is the methodology for carrying out the processes of retraining the reference models used in the method of multi-label classification of text messages dedicated directly to applications in the CC industry?
- RQ2: Which retraining strategy is more efficient for the CC industry—direct retraining of the existing network model using only new data or retraining the classifier with the full dataset, updated with newly acquired data?
- RQ3: Which retraining model has the best performance—models based on artificial neural networks ANN, multilingual BERT, monolingual HerBERT, or PolBERT?

The outline of the paper is as follows. Section “Research methodology” describes the methodology of the research conducted, while Section “Results” is a description of the results obtained and their analysis. The summary and conclusions are included in Section “Conclusion”.

## Research methodology

The main research work concerned the development and verification of the process of retraining the reference models used in the multi-label content classification method, implemented directly in a large CC system. The developed approach was verified based on^8^:

1. Retraining of models based on ANN and linear transformation methods: Principal Component Analysis (PCA), Latent Semantic Analysis (LSA), and Independent Component Analysis (ICA), which the authors developed for the problem of multi-label text message classification dedicated to applications in CC systems.
2. Retraining of multilingual BERT-type text data classification model and two BERT-type models dedicated directly to the Polish language: HerBERT, and PolBERT.

**Figure 1:**
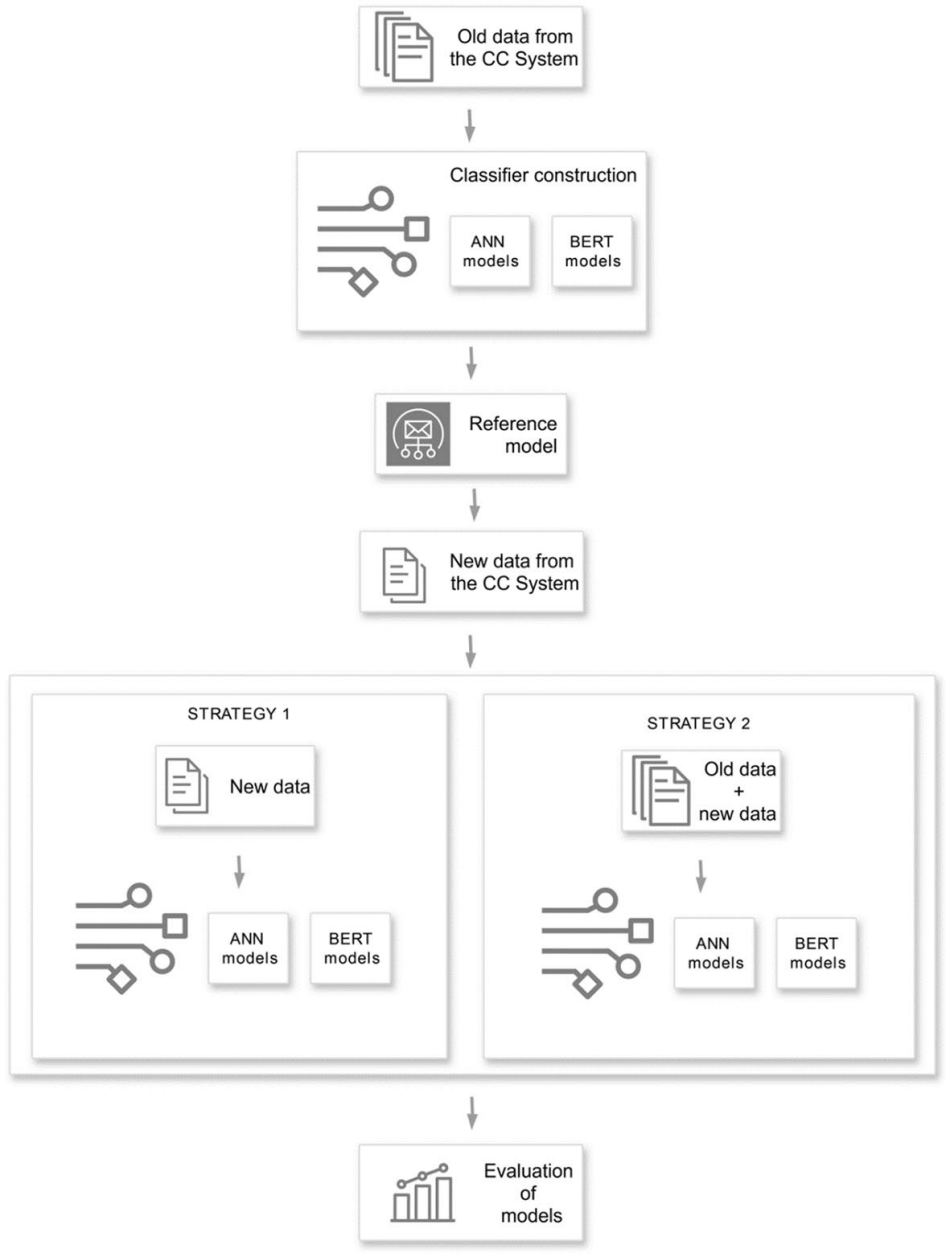
Diagram of the analyzed approach.

Figure 1 visualizes the approach used in the work. The first stage of the analyzed approach (classifier construction) is to construct the reference model based on old data. The analysis was based on Polish-language data from the actual archives of a large commercial contact center system (*CC System*) and English-language data extracted from a publicly available database (*Stackoverflow*). Both datasets were divided into two subsets: old data and new data. The old data accounts for 90% of all data and was used in the training of the reference models based on artificial neural networks (ANN-PCA, ANN-LSA, ANN-ICA) and based on BERT models (BERT, HerBERT, PolBERT).

The new data accounts for 10% of the total data and represents newly acquired data. The second stage of the approach is to retrain the reference models based on new data. We implemented two popular approaches to the problem of retraining artificial intelligence models. The first approach is a simpler technique and consists of direct retraining of the reference model using only new data. This way of conducting research is referred to hereafter as STRATEGY 1.

The second technique of retraining is more complex and involves retraining the reference models with the full dataset (including the old data and new data). This way of conducting research is referred to hereafter as STRATEGY 2.

The comparative analysis of both strategies was carried out with the use of k-fold cross-validation. The new data was divided into k subsets (folds). In each simulation run, one subset was used as validating data to verify the reference model and the retrained models, while the remaining subsets were used as training data. In STRATEGY 1, these subsets were used to retrain the reference model, while in STRATEGY 2, models were trained based on old data combined with subsets obtained from corresponding folds of new data.

The reference model and models retrained with the use of STRATEGY 1 and STRATEGY 2 were evaluated by calculating the arithmetic mean and standard deviation from k simulations (Evaluation of models). Learning parameters for the classifiers were selected using the grid-search-based method.

The following sections describe in detail the data used, the analyzed classification models, the criteria of evaluation of the classifiers, and the research environment developed.

### Dataset

The research works used two various datasets. The first dataset labeled *CC System* contains 10,301 records (data in Polish). Each record can belong to one or more classes related to the competencies of the employees: Incident, Service, ACC, ECM, SARA, and Systemic. The second dataset labeled *Stackoverflow* contains 10,000 records (data in English). Each record may belong to one or more classes from the set: Android, C#, C++, Java, Javascript, and Python. Both datasets therefore enable multi-label classification of text data. Each dataset was divided into two subsets: (1) the old data (used during the training and validation of reference models) comprising a total of 90% of all records, and (2) the new data used during the retraining and validation process comprising the remaining 10% of the data. The old *CC System* data contains 483,452 total words, including 86,393 unique words. The new *CC System* data contains 39,185 total words, including 13,220 unique words. The old *Stackoverflow* data contains 1,555,090 total words, including 167,263 unique words. The new *Stackoverflow* data contains 175,496 total words, including 27,329 unique words. Table 1 contains the characteristics of the data used during the study, along with a description of the different types of classes identified and their numbers. The *CC System* dataset was selected from actual databases of a large commercial CC system. The *Stackoverflow* dataset was extracted from a publicly available database^14^.

**Table 1:** Used data.

Dataset name	Date volume		Class type	Class size	
	Rd [pcs]	Nd [pcs]		Rd [pcs]	Nd [pcs]
*CC system*	9271	1030	Incident	6108	693
			Service	3125	324
			ACC	4252	458
			ECP	1779	183
			SARA	2637	301
			Systemic	439	47
*Stackoverflow*	8999	1001	Android	1558	160
			C#	1551	178
			C++	1565	179
			Java	1549	163
			Javascript	1521	181
			Python	1559	165

*Rd* Reference data, *Nd* New data.

**Figure 2:**
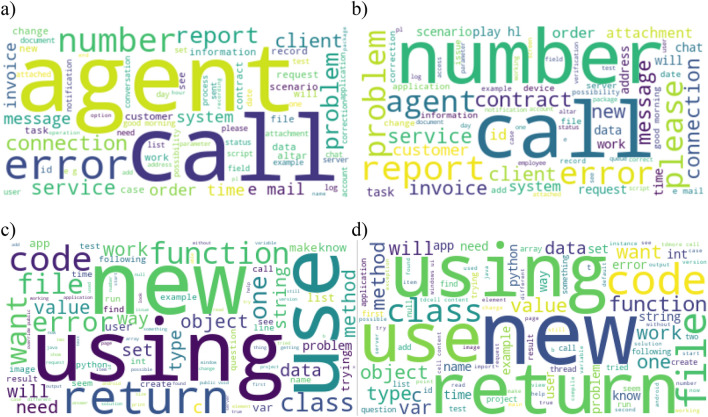
Word cloud with the most frequent words (**a**) for the old *CC System* data, (**b**) for the new *CC System* data (**c**) for the old *Stackoverflow* data, (**d**) for the new *Stackoverflow* data.

**Figure 3:**
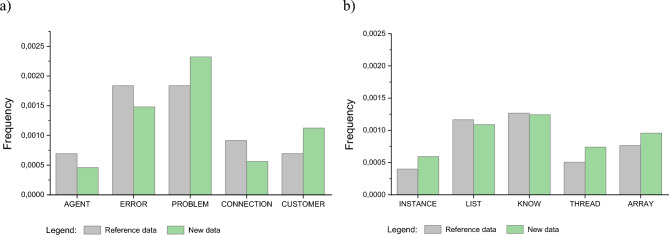
Frequency of selected words: (**a**) for the *CC System* data, (**b**) for the *Stackoverflow* data.

Figure 2a shows the word cloud of the most common words in the old *CC System* data, Fig. 2b shows the cloud for the new *CC System* data, Fig. 2c shows the word cloud of the most common words in the old *Stackoverflow* data, and Fig. 2d shows the cloud for the new *Stackoverflow* data. As can be seen from the analysis of the figure with the new data, the frequency of occurrence of individual words has changed. Figure 3 shows the differences in the frequency of selected words in old data and in new data. Moreover, the new data contains words with a completely new meaning that were not in the old data. For example, for the CC System dataset, there were new words such as: termination, subtitles, informal, among, installation, appropriate, and involvement. For the *Stackoverflow* dataset, there were new words such as: warmed, permutable, jobservice, omitting, monotonically, and casual. Therefore, it can be assumed that such a situation will affect the effectiveness of the classification process.

The values presented in the figure indicate the frequency ratio, which represents the number of occurrences of a given word relative to the total number of words collected from the dataset under study.

### Classification models

This section details the manner of configuration of the classification models used in the study. (a) classifiers based on ANN, (b) classifiers based on BERT-type models are described subsequently.

#### Classifiers based on ANN

The ANN-based model analyzed in the paper uses a hybrid combination of vectorization, dimensionality reduction, and classification methods. The approach takes into account tokenization, which consists of dividing text data (messages) into a list of words (tokens), taking into account the filtering of marginal words included in the so-called stop list^15^. Popular techniques based on word frequency were used to convert text data into numerical form: Count Vectorizer (CV), Term Frequency Vectorizer (TF), Term Frequency – Inverse Document Frequency Vectorizer (TF-IDF), and the ability to represent combinations of words (n-grams) in a vector: Bag on n-Grams (BoG)^16^. For dimensionality reduction, linear space transformation methods have been used to map vectors from one vector space to vectors in another space^17^. The following approaches were analyzed: LSA, PCA, and ICA. From the area of classification methods, an ANN of the multilayer perceptron type was used. The analysis of the classifier based on ANN was carried out for three selected architectures that obtained the best classification results presented in the paper^9^. The TF-IDF method was used to vectorize the data, including the use of single occurrences of words (unigrams) and combinations of two words (bigrams). Each architecture uses a different linear space transformation method: the first model is based on the PCA method and is denoted hereafter as ANN-PCA, the second model is based on the LSA method denoted hereafter as ANN-LSA, while the third model is based on the ICA method denoted ANN-ICA. All three architectures are based on a single ANN with a structure containing 3 hidden layers with a number of neurons equal to respectively: 100, 100, 20, and with relu-type activation function. The model contains one output layer with 6 neurons and a sigmoidal activation function. In addition, a dropout parameter = 0.2 was used, responsible for randomly discarding inputs to prevent overfitting. The models were trained using the Old Data set and the Adam algorithm for the following parameters: learning rate LR = 0.0001, batch size BS = 200, number of epochs EN = 500.

#### Classifiers based on BERT-type models

The paper also examines an approach to natural language representation based on a BERT-type model. Models of this type are trained in two stages. The first stage is pre-training, during which the model is trained on huge corpora of unlabeled text data. The second phase is fine-tuning, in which the BERT model is first initialized with pre-trained parameters, and then the parameters are adjusted to solve a specific problem, such as classifying text data in a specific subject area^13^. The original BERT models were dedicated to the English language. The corpora used in the phase were the BooksCorpus^18^ and English Wikipedia. BERT-type models have become a popular tool used by researchers to classify text data due to their high efficiency. In^19^, the BERT model was used for multi-class classification of radiology reports. A hierarchical BERT model with an adaptive tuning strategy was proposed in^20^. In^21^, the use of a hybrid approach combining BERT with LSTM to binary classify news articles into fake or legitimate was analyzed.

Recently, two BERT-type architectures have been proposed in the literature: the Polbert model and the HerBERT model. These models were dedicated to applications with the Polish language, which was a great advantage from the point of view of practical applications of the multi-label text message classification method developed by the authors.

The PolBERT model was developed in 2020^11,12^. It is available in two variants: cased and uncased, however, according to the author's recommendation, the cased versions were used in this paper. The cased version of the model was pre-trained on the following corpora: Polish subset of Open Subtitles, Polish subset of ParaCrawl, Polish Parliamentary Corpus, and Polish Wikipedia.

The HerBERT solution is a series of BERT models trained to analyze textual data in Polish^22^. HerBERT was pre-trained based on two collections. The first dataset consists of the National Corpus of Polish Language (NKJP) corpus, Wikipedia, and Free Readings. The second dataset additionally includes CCNet Head, CCNet Middle, and Open Subtitles texts. The HerBERT model uses Byte-Pair Encoding tokenization. In addition, BPEDropout was applied with a call rejection probability of 10%. HerBERT is a multilayer bidirectional transformer.

In the papers^10,22^, the models were verified using the standard KLEJ Benchmark model evaluation method. The research presented in^18^ confirms the superiority of HerBERT models over other popular models for Polish: Polish RoBERT and XLMRoBERT. In addition to the BERT approach, this paper also analyzed the POS Tagging and Dependency Parsing task.

All BERT-type models used in our research (BERT, PolBERT, HerBERT) are the basic models with 12 layers, 12 attention heads, and a hidden dimension of 768 (base).

### Evaluation of models

The research work focused on determining the feasibility of using the indicated manners of retraining as components of the multi-label content classification method developed by the authors. The main objective of the work carried out was to improve the quality of the developed classification method dedicated directly to CC systems supporting the Polish language. The work carried out ultimately allowed us to identify the mechanisms that best affect the quality of the primary models' retraining for the problem under consideration. Verification of the retraining process was realized through the use of multiple cross-validation. The quality of the classification of the learned models was determined using the most demanding metric used in multi-label classification known as an exact match, which is denoted hereafter by the symbol *Emotica*. *Emotica* is calculated as the percentage of correctly classified messages concerning the number of all messages. In the case of a message belonging to more than one class, the classification is considered correct when the method assigns the message correctly to all desired classes, according to formula (1):

1 $$Emotica= \frac{1}{n}\sum_{j=1}^{n}I(h\left({x}_{j}\right)={y}_{j})\cdot 100\%$$

where: $n$—the number of messages; $h\left({x}_{j}\right)$—the set of predicted labels for message; ${x}_{j}$, ${y}_{j}$—the set of true labels of message ${x}_{j}$; $I\left(true\right)=1; I\left(false\right)=0$

The second of the classification quality measures used was the *Accuracy* metric^23^. *Accuracy* was described as the ratio of the number of notifications in which a certain record was correctly classified by the system to the total number of notifications, according to formula (2).

2 $$Accuracy=\sum_{i=1}^{Q} \frac{{acc}_{i}}{Q}\cdot 100\%$$

where: $i=1\dots Q$ and $Q$—the number of classes; ${acc}_{i}$—is described as follows:

3 $${acc}_{i}=\frac{{TP}_{i}+{TN}_{i}}{{TP}_{i}+{FP}_{i}{+FN}_{i}+{TN}_{i}}$$

where: *TP*_*i*_—the number of records correctly classified from the i-th class; *TN*_*i*_—the number of records correctly unassigned to the i-th class; *FP*_*i*_—the number of records incorrectly assigned to the i-th class; *FN*_*i*_—the number of incorrectly classified records from the i-th class.

Additionally, due to unbalanced data, the F1score metric for individual classes was calculated for selected models. *F1score* represents the harmonic mean of *Precision* and *Recall* and can be calculated as follows^23^:

4 $${F1score}_{i}=\frac{2{TP}_{i}}{{2TP}_{i}+{FP}_{i}{+FN}_{i}}$$

where: *TP*_*i*_—the number of records correctly classified from the i-th class; *FP*_*i*_—the number of records incorrectly assigned to the i-th class; *FN*_*i*_—the number of incorrectly classified records from the i-th class.

### Research environment

**Figure 4:**
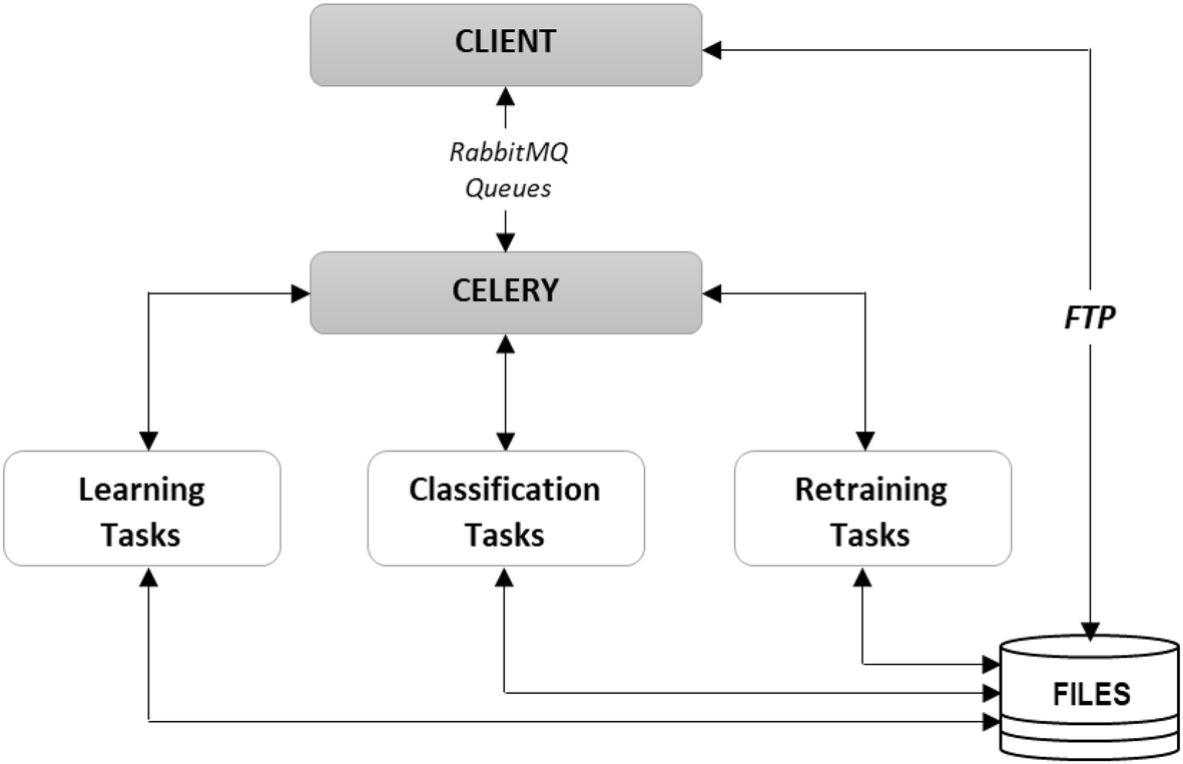
Illustrative diagram of how the test environment works.

For the purpose of the research works, a dedicated development environment and services were prepared to integrate the models under study with an external CC system. This made it possible to test different retraining approaches using data directly from a real system. Communication was implemented through RabbitMQ queues and *.csv type files. RabbitMQ is a message broker that implements the Advanced Message Queuing Protocol (AMQP), as well as other protocols. Its main features consist of message queuing, scalability, good integration with Celery, and good management and monitoring^24^. In the initial phase, the client imports files used for model retraining or data classification to a dedicated FTP server with a specific location. The client then sends a message with the FTP location to one of the RabbitMQ queues dedicated to the task: training, classifying, or retraining. The Celery service listens to the queues, and when a relevant message appears in one of them, a separate thread starts the corresponding task. Celery is a distributed task or job queue implemented in Python. It bases its operation on the use of so-called workers and clients. Its main features include real-time scheduling, synchronous and asynchronous task execution, and support for different languages. Additionally, Celery uses so-called message brokers, which are dedicated to facilitating communication between clients (which produce tasks) and workers (which consume tasks). Celery supports different message brokers, of which the most common and recommended are Redis and RabbitMQ^25^. The result of the task execution is sent back to the dedicated RabbitMQ queue for presentation to the client. The process of retraining for the previously described two variants was triggered dynamically by entering the relevant data for retraining. In the production version, such a service could be triggered, for example, when 100 misclassified records are registered in the system. The illustrative block diagram of the implemented test environment is presented in Fig. 4.

The tasks of training classifiers, data classification tasks, and retraining tasks were implemented through dedicated modules implemented in Python. Comparative analyses have been carried out for the ways of retraining proposed in the article using three selected models possible for the categorization of text data. A classifier based on an ANN network was implemented using the Scikit-learn Library^26,27^ and Keras Library^28,29^. The HerbBERT model was implemented using the HerBERT library^22^. The PolBERT model was implemented based on the PolBERT library^12^. Training algorithms and parameters were selected by trial and error for each classifier separately. Model learning and verification was carried out through a triple cross-validation process. The next section describes in detail the research results obtained.

## Results

During the course of the research conducted, the two strategies described in Section “Research methodology” used in the retraining issue for the three classification models selected were analyzed. The following sections describe the results obtained successively for: a classifier based on artificial neural networks (ANN) and linear space transformation methods (PCA, LSA, ICA), and BERT-type classifiers: the basic BERT model, the HerBERT model, and the PolBERT model. The obtained results of the retraining (STRATEGY 1, STRATEGY 2) in the form of mean values and standard deviation for the *Accuracy* and *Emotica* metrics were compared with the results obtained before retraining for the reference models. The reference model was built on old data and then evaluated on new data in order to compare the results obtained before and after retraining. A k-fold cross-validation process was conducted for each experiment. Given the number of records in the new data sets, the value of the parameter k was set to 3. Learning parameters were selected based on the grid-search-based method. For the classifiers based on artificial neural networks, the following values were analyzed:

- Batch size (BS) is selected from 1 to 200.
- Learning rate (LE) is selected from {0.0001, 0.0005, 0.001, 0.01}.
- Number of epochs (EN) is selected from 50 to 700.

For the classifiers based on BERT models, the following values were analyzed:

- Number of epochs (EN) is selected from 5 to 20.
- Max len (ML) is selected from {50, 100, 200, 300}.
- Learning rate (LE) is selected from {0.0001, 0.0002, 0.00001, 0.00002}.

### Experiments for the CC system dataset

This section summarizes the detailed results obtained for the CC System dataset containing data in the Polish language. In the first stage, models based on artificial neural networks and linear space transformation methods: ANN-PCA, ANN-LSA, and ANN-ICA, were analyzed. Table 2 summarizes the results of these experiments.

**Table 2:** Cross-validation results for classifiers based on the ANN model.

Model name	Reference model				STRATEGY 1				STRATEGY 2			
	Accuracy	SD	Emotica	SD	Accuracy	SD	Emotica	SD	Accuracy	SD	Emotica	SD
	[%]	[%]	[%]	[%]	[%]	[%]	[%]	[%]	[%]	[%]	[%]	[%]
Training data												
ANN-PCA	90.55	0.29	70.39	0.96	95.70	0.46	84.08	1.35	97.54	0.07	89.90	0.36
ANN-LSA	91.76	0.32	74.47	1.14	97.02	0.22	88.54	0.80	97.33	0.14	89.66	0.47
ANN-ICA	92.22	0.40	75.34	0.80	96.05	0.39	85.24	1.20	97.06	0.09	88.01	0.49
Testing data												
ANN-PCA	90.55	0.58	70.39	1.92	91.26	0.71	72.52	2.03	92.43	0.49	76.70	1.56
ANN-LSA	91.76	0.64	74.47	2.29	**93.02**	**0.51**	**77.38**	**1.28**	92.62	0.28	76.41	0.45
ANN-ICA	92.22	0.80	75.34	1.61	92.93	0.56	76.90	1.46	92.78	0.60	76.99	1.57

Significant values are in bold.

*SD* Standard deviation.

The best average accuracy of the reference model when studying training and testing data was obtained for the ANN-ICA solution. For the test set, the values of the *Accuracy* and *Emotica* metrics were 92.22 ± 0.4% and 75.34 ± 1.61%, respectively. In this case, the individual variable parameters were set as batch size BS = 200, epochs number EN = 500, and learning rate LR = 0.0001, respectively. For STRATEGY 1, the best average results were obtained using the model labeled ANN-LSA, for which *Accuracy* and *Emotica* for the testing data were 93.02 ± 0.51% and 77.38 ± 1.28%, respectively. The BS, EN, and LR parameters for which the best results were obtained in this case were: BS = 150, EN = 100, and LR = 0.0001. For STRATEGY 2, the best results for *Accuracy* = 92.78 ± 0.60% and *Emotica* = 76.99 ± 1.57 were obtained for the ANN-ICA model for the parameters: BS = 100, EN = 500, and LR = 0.0001. In terms of the *Emotica* metric for STRATEGY 2, the results for all models tested are fairly similar between 76 and 77%. However, the ANN-LSA model appears to be more stable in this case, for which standard deviation values were obtained at the lowest level of ± 0.45%. In this case, the relevant parameters are defined at respectively: BS = 200, EN = 700, and LR = 0.0001. In the case analyzed, apparently the best results were obtained for the ANN-LSA model, which was retrained according to the assumptions of STRATEGY 1. The results obtained for the *Emotica* metric in this case are more than 2% better than the reference model, which should be considered a significant improvement in classification quality. For this case, Table 3 shows the detailed results of the cross-validation process, from which it can be seen that during the test for fold 2, the classification efficiency of the test data was the highest, and was respectively: *Accuracy* = 93.63% and *Emotica* = 79.01%.

**Table 3:** Detailed cross-validation results for STRATEGY 1 and the ANN-LSA model (CC System dataset).

k	Training data		Testing data	
	Accuracy	Emotica	Accuracy	Emotica
	[%]	[%]	[%]	[%]
1	97.28	89.65	92.39	75.87
2	96.75	87.77	**93.63**	**79.01**
3	97.04	88.21	93.05	77.26
Average	97.02	88.54	93.02	77.38
SD	0.22	0.80	0.51	1.28

Significant values are in bold.

Table 4 shows the detailed results obtained for STRATEGY 2 for the ANN-ICA model. The learning parameters were respectively: BS = 100, EN = 500, and LR = 0.0001. The highest *Accuracy* = 93.54% and *Emotica* = 78.72% metrics were obtained during the test for fold No. 3.

**Table 4:** Detailed cross-validation results for STRATEGY 2 and the ANN-ICA model (CC System dataset).

k	Training data		Testing data	
	Accuracy	Emotica	Accuracy	Emotica
	[%]	[%]	[%]	[%]
1	97.18	88.19	92.73	77.33
**2**	97.02	88.50	92.08	74.93
3	96.97	87.34	**93.54**	**78.72**
Average	97.06	88.01	92.78	76.99
SD	0.09	0.49	0.60	1.57

Significant values are in bold.

The next stage of the research was to verify the effectiveness of the multilingual BERT model and two BERT-type models dedicated directly to the Polish language. Table 5 summarizes the results of cross-validation studies for the BERT models.

**Table 5:** Cross-validation results for BERT-type models.

Model name	Reference model				STRATEGY 1				STRATEGY 2			
	Accuracy	SD	Emotica	SD	Accuracy	SD	Emotica	SD	Accuracy	SD	Emotica	SD
	[%]	[%]	[%]	[%]	[%]	[%]	[%]	[%]	[%]	[%]	[%]	[%]
Training data												
BERT	90.92	0.56	71.12	1.81	99.61	0.02	97.96	0.12	98.74	0.14	94.56	0.70
PolBERT	92.01	0.40	73.74	1.63	99.86	0.02	99.22	0.18	99.62	0.08	97.96	0.43
HerBERT	92.35	0.66	75.00	1.91	99.62	0.06	97.96	0.43	98.86	0.47	94.81	1.42
Testing data												
BERT	90.76	0.84	70.87	2.14	93.24	0.60	77.09	2.16	92.06	0.60	74.66	1.64
PolBERT	91.94	1.2	73.79	4.33	93.61	0.80	78.06	2.03	92.51	0.49	75.05	1.57
HerBERT	92.01	0.69	74.17	2.17	**93.85**	**0.24**	**79.13**	**0.66**	93.61	0.90	78.55	2.46

Significant values are in bold.

*SD* Standard deviation.

Analyzing the results in Table 5, it can be seen that, as in the previous case, STRATEGY 1 performs better. For the HerBRET model, efficiency as measured by the *Emotica* metric is at 79.13 ± 0.66%, an improvement of less than 5% over the reference model. These values were obtained for the parameters ML = 200, EN = 10, LR = 0.00002. It can also be noted that in this case the stability of the HerBERT model is better than that of the PolBERT model and the multilingual BERT model, and the standard deviations of the analyzed metrics are several times lower. On the other hand, the *Accuracy* metric for both STRATEGIES and both models achieves a fairly similar value oscillating between 92.51% and 93.85%. Table 6 shows the detailed results of the cross-validation process for the HerBERT model retrained according to STRATEGY 1. The data shows that the highest classification accuracy of the test data was obtained for the second fold. Classification efficiency was *Accuracy* = 94.17% and *Emotica* = 79.59%, respectively.

**Table 6:** Detailed cross-validation results for STRATEGY 1 and the HerBERT model.

k	Training data		Testing data	
	Accuracy	Emotica	Accuracy	Emotica
	[%]	[%]	[%]	[%]
1	99.73	98.40	93.60	78.20
2	99.68	98.11	**94.17**	**79.59**
3	99.56	97.38	93.78	**79.59**
Average	99.62	97.96	93.85	79.13
SD	0.06	0.43	0.24	0.66

Significant values are in bold.

Table 7 presents the results of the cross-validation for the HerBERT model retrained according to STRATEGY 2. The highest values of the analyzed metrics: *Accuracy* = 94.95% and *Emotica* = 81.92% were obtained during tests conducted for fold 3.

**Table 7:** Detailed cross-validation results for STRATEGY 2 and the HerBERT model.

k	Training data		Testing data	
	Accuracy	Emotica	Accuracy	Emotica
	[%]	[%]	[%]	[%]
1	99.27	96.21	92.78	76.16
2	99.10	95.34	93.10	77.55
3	98.20	92.87	**94.95**	**81.92**
Average	98.86	94.81	93.61	78.55
SD	0.47	1.42	0.95	2.46

Significant values are in bold.

Table 8 shows the detailed results of the cross-validation process for the PolBERT model retrained according to STRATEGY 1. As in the HerBERT model, the highest classification accuracy of the test data was obtained for the second fold. Classification efficiency was *Accuracy* = 94.70% and *Emotica* = 80.76%, respectively.

**Table 8:** Detailed cross-validation results for STRATEGY 1 and the PolBERT model.

k	Training data		Testing data	
	Accuracy	Emotica	Accuracy	Emotica
	[%]	[%]	[%]	[%]
1	99.88	99.27	92.83	75.87
2	99.88	99.42	**94.70**	**80.76**
3	99.83	98.98	93.29	77.55
Average	99.86	99.22	93.61	78.06
SD	0.02	0.18	0.80	2.03

Significant values are in bold.

Table 9 presents the detailed cross-validation results for the PolBERT model retrained according to STRATEGY 2. The highest values of the analyzed metrics: *Accuracy* = 93.20% and *Emotica* = 77.26% were obtained during tests conducted for fold 3.

**Table 9:** Detailed cross-validation results for STRATEGY 2 and the PolBERT model.

k	Training data		Testing data	
	Accuracy	Emotica	Accuracy	Emotica
	[%]	[%]	[%]	[%]
1	99.56	97.52	92.25	74.13
2	99.73	98.54	92.08	73.76
3	99.56	97.82	**93.20**	**77.26**
Average	99.62	97.96	92.51	75.05
SD	0.08	0.43	0.49	1.57

Significant values are in bold.

**Figure 5:**
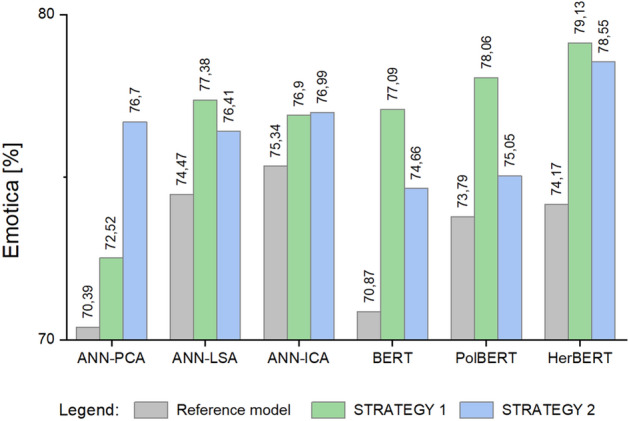
Mean values of the *Emotica* metric for the *CC System* dataset.

**Figure 6:**
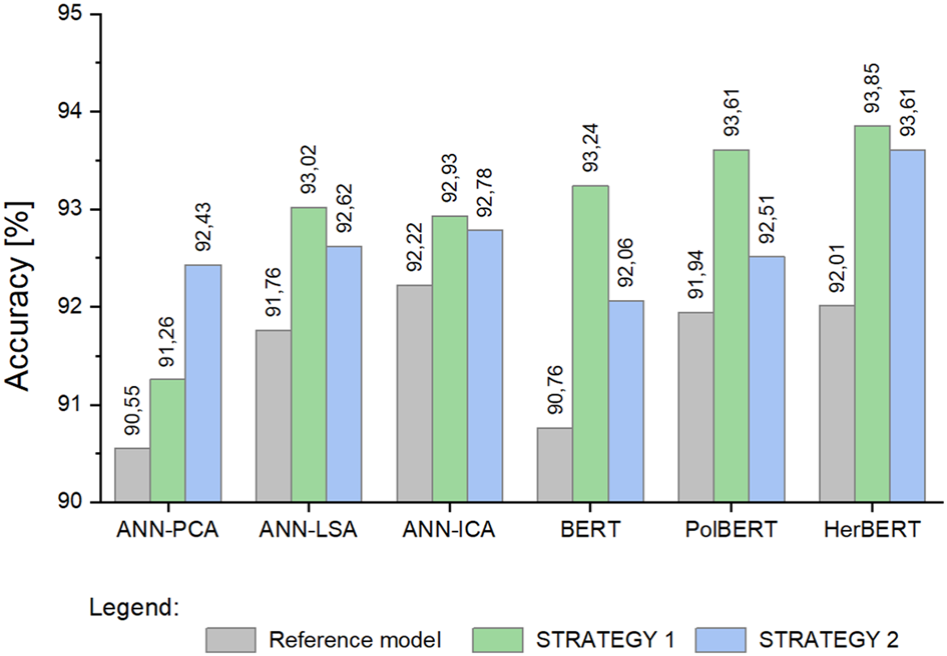
Mean values of the *Accuracy* for the *CC System* dataset.

Figure 5 shows a comparison of the obtained values for the *Emotica* metric tested for the different models discussed in Section “Experiments for the CC System dataset”. Figure 6 illustrates the results obtained for the *Accuracy* metric. The classification accuracy obtained before and after the application of the retraining according to STRATEGY 1 and according to STRATEGY 2 are presented. Of all the approaches compared, the highest values were obtained using the HerBERT model and STRATEGY 1.

**Figure 7:**
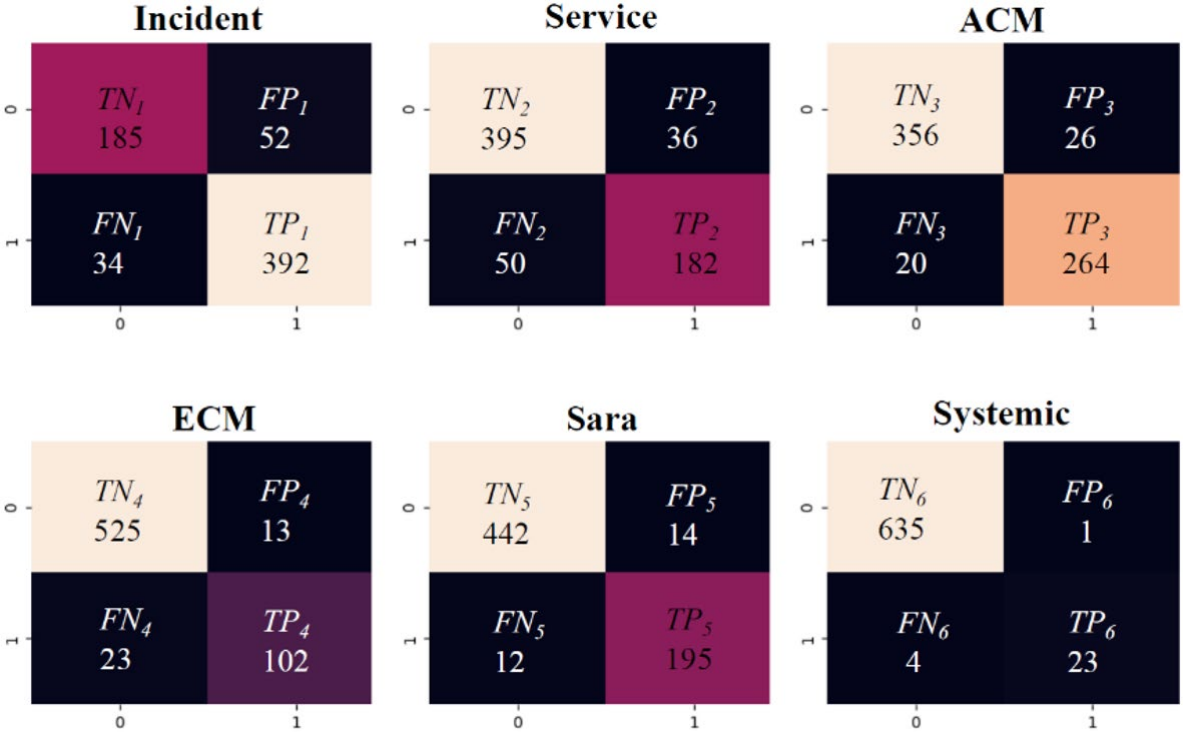
Confusion matrix for the ANN-LSA model according to STRATEGY 1 (CC System dataset).

In addition, Fig. 7 presents the confusion matrices obtained for an example model based on artificial neural networks and the LSA method and STRATEGY 1. This matrix presents the number of correctly classified records in each of the 6 classes analyzed. Table 10 shows the detailed results of *Accuracy* and *F1score* obtained for each class based on testing data.

**Table 10:** Results of *Accuracy* and *F1score* obtained for each class based on testing data (CC System dataset).

Class	Testing data	
	Accuracy	F1 score
	[%]	[%]
Incident	87.03	90.11
Service	87.03	80.89
ACM	93.09	91.99
ECM	94.57	85.00
Sara	96.08	93.75
Systemic	99.25	90.20

### Experiments for the Stackoverflow dataset

This section presents the results obtained for the Stackoverflow dataset containing data in the English language. Models based on artificial neural networks and linear space transformation methods: ANN-PCA, ANN-LSA, ANN-ICA, and the multilingual BERT model were analyzed. Table 11 summarizes the results of these experiments.

**Table 11:** Cross-validation results for classifiers based on the ANN model and the BERT model.

Model name	Reference model				STRATEGY 1				STRATEGY 2			
	Accuracy	SD	Emotica	SD	Accuracy	SD	Emotica	SD	Accuracy	SD	Emotica	SD
	[%]	[%]	[%]	[%]	[%]	[%]	[%]	[%]	[%]	[%]	[%]	[%]
Training data												
ANN-PCA	93.62	0.18	77.68	0.49	97.47	0.34	89.47	1.30	98.88	0.09	94.92	0.33
ANN-LSA	95.35	0.19	82.99	0.87	97.82	0.03	90.82	0.40	99.45	0.02	97.08	0.28
ANN-ICA	95.50	0.10	83.44	0.31	96.01	0.03	84.65	0.22	97.72	0.14	90.46	0.71
BERT	96.27	0.10	86.32	0.59	99.90	0.07	99.45	0.35	99.77	0.04	98.70	0.28
Testing data												
ANN-PCA	93.62	0.37	77.68	0.99	93.97	0.52	78.58	1.74	93.97	0.32	79.03	0.70
ANN-LSA	95.35	0.39	82.98	1.74	95.38	0.48	82.72	1.34	95.38	0.54	83.79	1.77
ANN-ICA	95.50	0.20	83.44	0.62	95.68	0.28	83.71	0.89	95.52	0.21	83.44	1.10
BERT	96.54	0.16	87.41	0.24	**96.56**	**0.35**	**87.72**	**0.41**	96.49	0.44	86.92	2.06

Significant values are in bold.

*SD* Standard deviation.

The best values of *Accuracy* and *Emotica* were obtained for the BRET model for the parameters ML = 200, EN = 10, LR = 0.00002, and STRATEGY 1. Table 12 presents the results of the cross-validation for the sample ANN-LSA model, which was retrained according to the assumptions of STRATEGY 1. The highest values of the analyzed metrics: *Accuracy* = 96.14% and *Emotica* = 86.25% were obtained during tests conducted for fold 1.

**Table 12:** Detailed cross-validation results for STRATEGY 1 and the ANN-LSA model (Stackoverflow dataset).

k	Training data		Testing data	
	Accuracy	Emotica	Accuracy	Emotica
	[%]	[%]	[%]	[%]
1	99.44	97.03	**96.14**	**86.25**
2	99.42	96.76	95.00	82.16
3	99.48	97.44	95.00	82.97
Average	99.45	97.08	95.38	83.79
SD	0.02	0.28	0.54	1.77

Significant values are in bold.

Table 13 presents the results of the cross-validation for the sample BERT model, which was retrained according to the assumptions of STRATEGY 1. The highest values of the analyzed metrics: *Accuracy* = 97.05% and *Emotica* = 88.29% were obtained during tests conducted for fold 3.

**Table 13:** Detailed cross-validation results for STRATEGY 1 and the BERT model.

k	Training data		Testing data	
	Accuracy	Emotica	Accuracy	Emotica
	[%]	[%]	[%]	[%]
1	99.80	98.95	96.26	87.43
2	99.95	99.70	96.36	87.43
3	99.95	99.70	**97.05**	**88.29**
Average	99.90	99.45	96.56	87.72
SD	0.07	0.35	0.35	0.41

Significant values are in bold.

**Figure 8:**
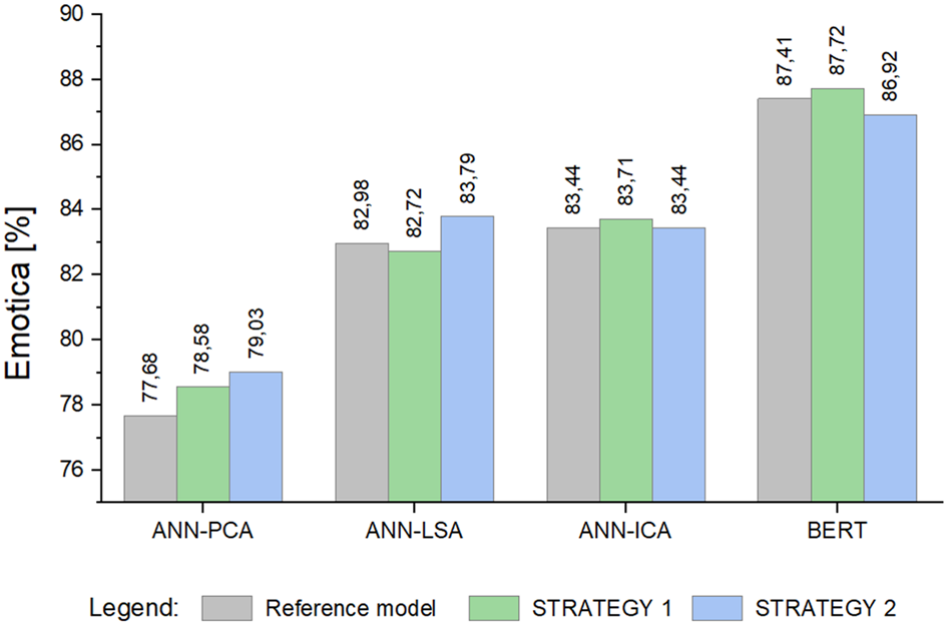
Mean values of the *Emotica* metric for the *Stackoverflow* dataset.

**Figure 9:**
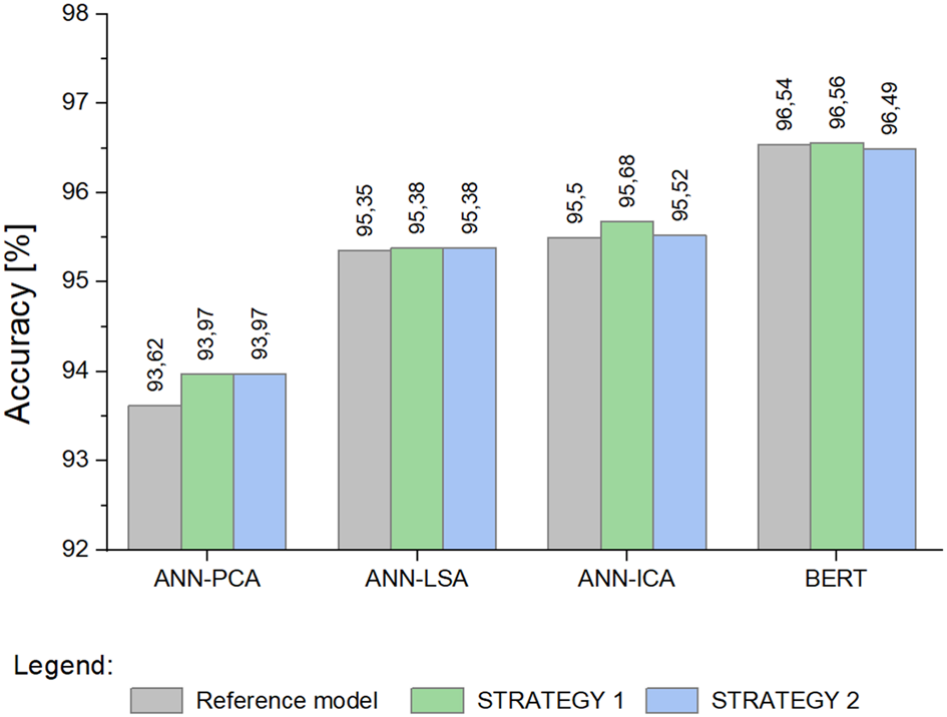
Mean values of the *Accuracy* metric for the *Stackoverflow* dataset.

Figure 8 shows a comparison of the obtained values for the *Emotica* metric. Figure 9 illustrates the results obtained for the *Accuracy* metric. Of all the approaches compared, the highest values were obtained using the BERT model and STRATEGY 1.

**Figure 10:**
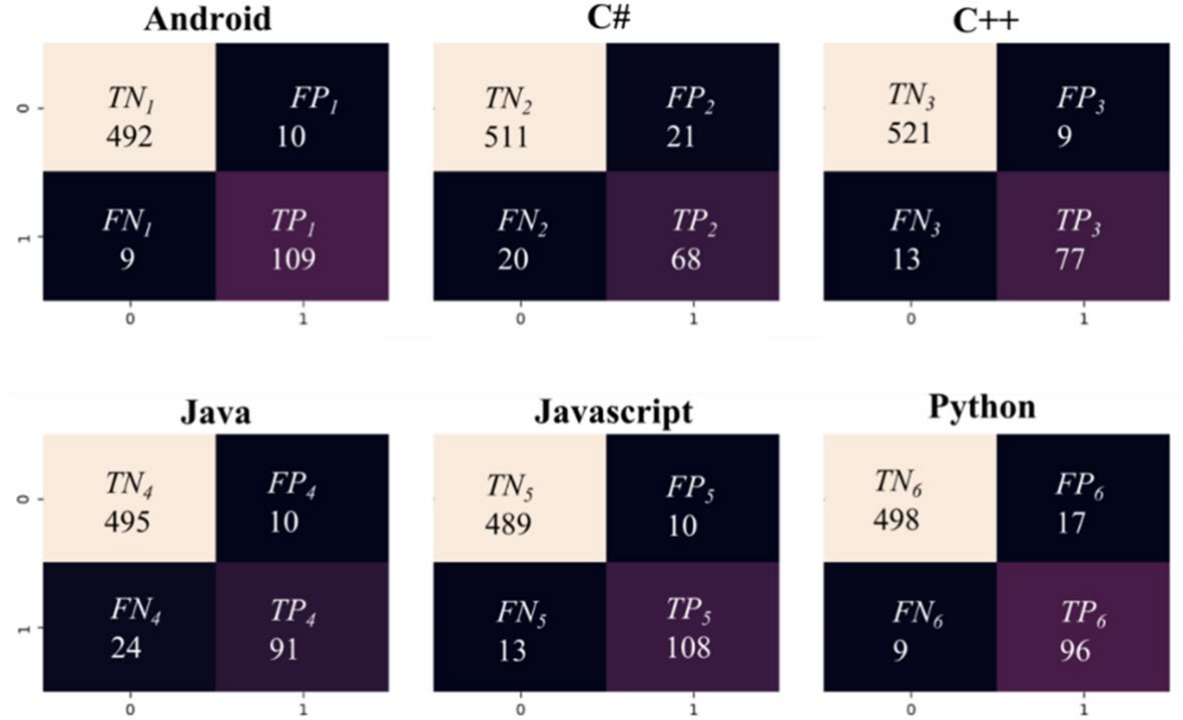
Confusion matrix for the ANN-ICA model according to STRATEGY 1 (Stackoverflow dataset).

In addition, Fig. 10 presents the confusion matrices obtained for an example model based on artificial neural networks and the LSA method and STRATEGY 1. This matrix presents the number of correctly classified records in each of the 6 classes analyzed. Table 14 shows the detailed results of *Accuracy* and *F1score* obtained for each class based on testing data.

**Table 14:** Results of *Accuracy* and *F1score* obtained for each class based on testing data (Stackoverflow dataset).

Class	Testing data	
	Accuracy	F1score
	[%]	[%]
Android	96.94	91.98
C#	93.39	76.84
C++	96.45	87.50
Java	94.52	84.26
Javascript	96.29	90.38
Python	95.81	88.07

## Conclusion

The paper analyzes the feasibility of two different strategies for the retraining of multi-label text data classifiers dedicated to the Call/Contact Centers industry. A comparison was made between the strategy of retraining the reference classification model with new data and updating the classifiers using the old data and the new data. The analysis was carried out using data in Polish from the actual archives of a large commercial CC system and using data in English from the publicly available dataset. A comparative analysis was carried out for three selected models based on ANN and linear space transformation methods, a multilingual BERT model and two BERT-type models developed for the Polish language: PolBERT and HerBERT. The quality of the classification of the learned models was assessed using the *Emotica* metric for determining exact fit, and the popular *Accuracy* metric. Additionally, the *F1score* metric was analyzed for the selected models.

In most of the simulations, higher values for both metrics analyzed were obtained for the strategy of retraining the reference classification model with the newly collected data. Each of the analyzed models obtained an increase in the values of the analyzed metrics both after applying STRATEGY 1 and also after retraining with STRATEGY 2. Of all the classifiers compared based on Polish data, the highest values were obtained for the HerBERT model. The *Emotica* metric for this model and STRATEGY 1 achieved an average value of 79.13 ± 0.66%, an improvement of about 5% over the reference model. Values of the analyzed metrics both after applying STRATEGY 1 and also after retraining with STRATEGY 2. Of all the classifiers compared based on English data, the highest values were obtained for the BERT model. The *Emotica* metric for this model and STRATEGY 1 achieved an average value of 87.72 ± 0.41%. The results confirm the validity of this approach in CC-type systems.

The retraining strategies analyzed in the paper have the following advantages:

- BERT models, which are large language models, allow for higher classification accuracy. However, the application of simpler models based on multi-layer perceptron artificial neural networks also yields satisfactory classification results.
- The research has shown that STRATEGY 1, which is a simpler and faster technique, allows for similar or in most cases better classification results after retraining compared to the more complex STRATEGY 2.
- The obtained results can be utilized in practical and commercial text data classification systems in CC-type companies and facilitate the choice of retraining classifier strategies.

The proposed solution in the paper has the following limitations:

- To maintain the effectiveness of the proposed method in campaigns with different themes than those presented in the paper, it is necessary to thoroughly prepare text data for classifier training processes. It should be emphasized that this is a time-consuming process that significantly affects the costs of potential new solution deployments.
- In the case of implementing the proposed solution for algorithms used in CC systems, which rely on data from transcription of audio conversations, it is necessary to additionally consider both the effectiveness of the ASR system and the quality of the audio data. In real-world conditions, environmental disturbances such as various types of noise can affect transcription quality, and consequently, the quality of retraining.
- Similar problems may arise with written text samples (chats, email messages), for example, when a person simplifies the message due to time pressure, leading to syntactical and grammatical errors, which can also affect the quality of the retraining process

As a direction for further research, it is planned to expand the study to include the possibility of using other retraining techniques. There are also plans to acquire new text datasets in Polish. The obtained results can be applied to other similar languages and languages with limited resources. Therefore, research is also planned for languages related to Polish. This includes, among others, the group of Slavic languages, which are similar both in terms of grammar and vocabulary. Additionally, studies are being considered for selected languages with limited data resources, including, for example, the Serbian language^30–33^.

## Data Availability

The data that support the findings of this study are available from the corresponding author upon reasonable request. Restrictions apply to the availability of these data, which were used under license from the Altar Sp. z o. o. company for the current study, and so are not publicly available.
